# German Translation and Validation of the Cognitive Style Questionnaire Short Form (CSQ-SF-D)

**DOI:** 10.1371/journal.pone.0149530

**Published:** 2016-03-02

**Authors:** Quentin J. M. Huys, Daniel Renz, Frederike Petzschner, Isabel Berwian, Christian Stoppel, Helene Haker

**Affiliations:** 1 Translational Neuromodeling Unit, Institute for Biomedical Engineering, University of Zürich and Swiss Federal Institute of Technology (ETH) Zürich, Zürich, Switzerland; 2 Centre for Addictive Disorders, Department of Psychiatry, Psychotherapy and Psychosomatics, Hospital of Psychiatry, University of Zürich, Zürich, Switzerland; 3 Department of Psychiatry and Psychotherapy, Charité Campus Mitte, Charité Universitätsmedizin, Berlin, Germany; University of Geneva, SWITZERLAND

## Abstract

**Background:**

The Cognitive Style Questionnaire is a valuable tool for the assessment of hopeless cognitive styles in depression research, with predictive power in longitudinal studies. However, it is very burdensome to administer. Even the short form is still long, and neither this nor the original version exist in validated German translations.

**Methods:**

The questionnaire was translated from English to German, back-translated and commented on by clinicians. The reliability, factor structure and external validity of an online form of the questionnaire were examined on 214 participants. External validity was measured on a subset of 90 subjects.

**Results:**

The resulting CSQ-SF-D had good to excellent reliability, both across items and subscales, and similar external validity to the original English version. The internality subscale appeared less robust than other subscales. A detailed analysis of individual item performance suggests that stable results could be achieved with a very short form (CSQ-VSF-D) including only 27 of the 72 items.

**Conclusions:**

The CSQ-SF-D is a validated and freely distributed translation of the CSQ-SF into German. This should make efficient assessment of cognitive style in German samples more accessible to researchers.

## 1 Introduction

Cognitive interpretations of events are paramount to the generation, maintenance and regulation of affect [1, 2]. Their measurement has relied heavily on two sets of questionnaires: initially the dysfunctional attitudes scale (DAS; [3]) and thereafter the attributional style questionnaires (ASQ; [4, 5]), which in turn was redeveloped into the cognitive style questionnaire (CSQ; [6]).

Both the ASQ and CSQ were developed in the context of hopelessness theories of depression [7] to probe how negative events are explained by individuals, specifically whether negative events are ascribed to global, internal and stable and positive events to unstable, external and specific causes. They have proven sensitivity to a history of depressive episodes and as a measure of risk for the development of future depressive episodes [6, 8].

Both ASQ and CSQ are very long and hence difficult to administer. [9] therefore produced a short form of the CSQ (CSQ-SF). Briefly, in the CSQ-SF, subjects are first asked to imagine particular scenarios, for instance ‘Imagine that you go to a party and people are not interested in you’. They are then asked asked to indicate their agreement with nine statements on a 5-point Likert scale. Two statements probed the internality of attributions (’It is not my fault that people are not interested in me.’), two their globality (’The reason people weren’t interested in me at this party will cause people at parties in the future not to be interested in me.’), two their stability (’If I go to a party like this in the future, things will be different and people will be interested in me.’), two their self-worth (’People not being interested in me at this party says a lot about me as a person.’) and one item assessed their judgement about likely negative consequences (’People not being interested in me at this party will not lead to other negative things happening to me.’).

While the CSQ-SF substantially reduces the burden on participants, it still involves rating 72 rather complex items which can easily take 30 minutes and hence continues to place a high burden on subjects. Although [9] examined reduced versions by removing entire scenarios, they did not perform an item-based analysis.

The measurement of cognitive factors is of great importance to theories of depression and to depression research. As such, it is important to translate the measures for use in other languages. Only the ASQ [10], but neither the CSQ nor its more easily administered short version have as yet been translated into or validated in German.

The current paper provides a translation of the CSQ-SF into German (CSQ-SF-D) with a validation of its reliability and factor structure and examination of external validity in a convenience sample of healthy participants. Principal component analyses (PCA) of the CSQ-SF indicated that it measured a single underlying dimension, akin to the CSQ. With the aim of further shortening it, we therefore performed additional item-based analyses and examine the resulting very short form (CSQ-VSF-D).

## 2 Methods

### 2.1 Translation

Four independent translations into German by native German speakers proficient in English were obtained. Three of these were clinically qualified (two psychiatrists, one psychologist). These were used to construct a first version of the questionnaire by the authors. This first version was commented on by four further, independent psychiatrists who were native German speakers fluent in English. These also had access to the original English version. Comments were included by QH to form the second version. This was then sent to a native English speaker proficient in German for backtranslation, and the backtranslation compared to the English original by two further native speakers. These comments were again considered by QH and only minor further changes implemented.

During the translation, we altered two items to make them more applicable to general populations rather than student populations. The setting of items 2 and 6 was changed to a continuing education setting rather than a university or school setting. To reduce response set bias, we attempted to stay close to the original version by [9] and did not formulate the questions according to a rigid schema.

The questions in the final version of the questionnaire are reproduced in the appendix. These questions were implemented as an online version using LimeSurvey (www.limesurvey.org).

### 2.2 Test population

Subjects were recruited through email adverts through the University of Zürich recruitment system (UAST). The study was approved by the local ethics committee (Kantonale Ethikkommission Zurich). After providing informed consent, 214 subjects filled in the short version of CSQ questionnaire online via a customized version of LimeSurvey. Of these, 206 were complete. Subjects additionally reported measures of obsessive-compulsive traits (PADUA-Inventory Washington State University Revision (PI-WSUR); [11], German translation: University Clinic Bonn (2002)) as well as impulsivity (Barrat Impulsiveness Scale (BIS-11); Original: [12], German translation: [13]).

A subset of 90 participants took part in a follow-up study. For this convenience subset the following additional questionnaires were acquired: the German version of the Penn State Worry Questionnaire (PSWQ-D, Original: [14], German Translation: [15]), Sensitivity to Punishment and Reward Questionnaire (SPSRQ, [16]), Stait-Trait Anxiety Inventory (STAI, [17]), Frost Multidimensional Perfectionism Scale (FMPS-D, [18], German Translation: [19]), Inventory of Depressive Symptomatology Self Report (IDS-SR30, [20]) and Obsessive Compulsive Inventory-Revised (OCI-R, [21], German Translation: [22]). In addition, these subjects performed learning and decision-making tasks aimed at assessing processes underlying obsessive and compulsive symptoms in healthy participants. These are not reported here as we had no a priori hypotheses about their relationship with CSQ measures; and as it would detract from the focus of the paper, which is the establishment and validation of the CSQ in German.

### 2.3 Analysis

All analyses were performed using Matlab 8.4.0.150421 (R2014b). Analyses were performed on individual items; on the five pre-defined subscales (internality, items 1 and 6; globality, items 2 and 7; stability, items 3 and 8; negative consequences, item 4; self-worth, items 5 and 9 of each scenario), and on the scenarios.

Cronbach *α* was calculated across items, across subscales and across scenarios to measure internal reliability of the scores. Pearson’s correlations were measured between subscales and with the total scores.

We performed a principal component analysis (PCA) without preprocessing of the data (by computing the eigenvalues of the covariance matrix). Variance explained was measured as the normalised eigenvalue. PCAs were performed on individual items, on subscales and on scenarios.

In addition to PCA, we also performed a factor analysis using the function factoran.m. BIC scores were computed as follows:

$$\begin{array}{c} {\text{BIC}}_{k}=-2L+\left(jk+j-\frac{k(k-1)}{2}\right)\log\left(N\right) \end{array}$$

where L is the total likelihood, *k* is the number of factors in the model, *j* the dimensionality of each subject’s data (*j* = 72 for factor analyses on individual items, and *j* = 5 for factor analyses across subscales) and *N* the number of subjects.

We additionally examined the linear correlation of each individual item, subscale and scenario with the total score. The linear correlation factors were then ordered. We assessed the correlation of the sum across the *n* most correlated items, subscales or scenarios with the total scores to look at how many items were necessary to reach a correlation of 0.95. This was performed once in the entire dataset, and once in two random partitions of the datasets. Finally, the ordering of the items/subscales/scenarios in the half datasets and the full dataset were compared.

## 3 Results

The full set of final items is displayed in S1 File. The full data is contained in S2 File. As in the original, total scores could range from 72 to 360. 206 full answer sets were collected. Sample characteristics are presented in Table 1, and the correlations between the five subscales internality, globality, stability, self-worth, negative consequences and the total scores are shown in Table 2. As can be seen, the internality score was both less correlated with the other scores, and also less correlated with the total score.

**Table 1:** Sample characteristics.

	Mean	St. Dev	Cronbach’s *α*
Age	23.8	3.2	
Male	34%		
CSQ-SF total score	185.5	27.3	0.91
Internality	48.9	5.4	0.48
Globality	37.2	7.2	0.69
Stability	41.3	7.8	0.73
Self-worth	41.4	10.2	0.82
Negative consequences	16.7	5.3	0.65

**Table 2:** Subscale correlations.

	Globality	Stability	Self-worth	Negative Consequences	Total
Internality	0.16*	0.22*	0.31*	0.07	0.43*
Globality		0.71*	0.63*	0.59*	0.85*
Stability			0.59*	0.51*	0.84*
Self-worth				0.45*	0.86*
Negative Consequences					0.68*

Significant linear correlations *p* <.05 are denoted by *.

### 3.1 Item-based measures

**Fig 1:**
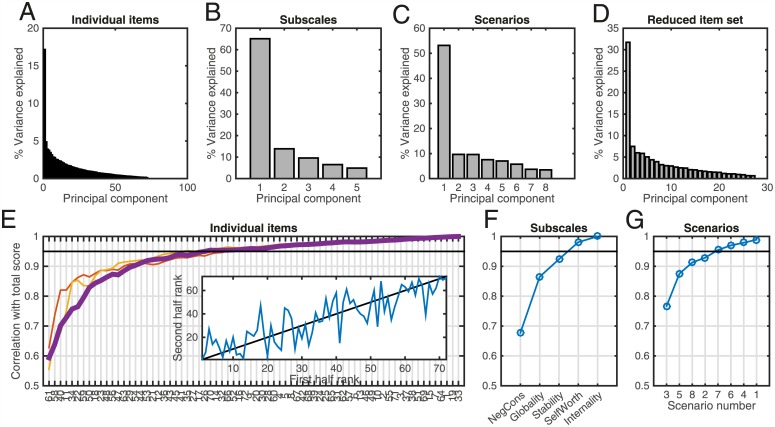
Component structure of the CSQ-SF-D. **A-D**: PCA results: variance explained (normalised eigenvalues) across individual items (A), across the five subscales (B), across the eight scenarios (C) and across the reduced set of 27 items for the very short form (E). **E**: Correlations of subscales composed of increasing number of items highly correlated with total score. The leftmost point shows the correlation of the most predictive item (61). Going towards the right, the second-most predictive item is added, and the correlation of the sum across these two items with the total score is shown. As the most informative items are added, the correlation with the total score increases rapidly and then flattens of. The level of 0.95 correlation is reached at around 27 items in the full dataset (thick line), and also in the two split-half datasets (thin lines). **Inset**: Individual item rankings in terms of correlations with total score are similar across the two halves of the dataset (correlation = 0.84, *p* < 1^−20^). **F, G** Analysis as in E, but across subscales (F) and scenarios (G).

Overall, when considering the sum of all individual items, Cronbach’s *α* indicated excellent reliability (*α* = 0.91), and a principal component analysis suggested the existence of one single underlying factor (Fig 1A) explaining 17% of the variance. The factor loadings were spread evenly (all but 5 items loading positively), suggesting that variation was best captured by the total score. The factor analysis also indicated that the best fit contained only one hidden factor (BIC = 817 for one factor compared to 1191 for two factors).

Given that the CSQ-SF-D measured a single underlying dimension, rather equally across items, and that the extremely high reliability indicated possible redundancy, we examined how individual items predicted the total score. To do so, we measured the linear correlation between each item and the total score. We then asked how well one could predict the total score by incrementally including items that individually correlated most strongly with the total score. Fig 1D shows the results of this analysis. The best-performing item (number 61, item 7 in scenario 6) on its own had a correlation of 0.6 with the total score. As more items are included, the correlation with the total score increases rapidly, reaching 0.95 when around 27–30 of the 72 items are included. This was true when doing the analysis in the entire dataset (thick blue line), and remained stable when repeating the analysis in the two halves of the dataset (thin lines). The rankings inferred in each of the two halves of the dataset were also highly correlated (Fig 1D, inset).

The 27 most informative items (Table 3) were distributed across scenarios 2–7, with no item from scenario 1 or 8. They were also spread across all the subscales (7 items measuring globality; 7 items measuring stability; 10 items measuring self-worth and 3 items measuring negative consequences) except for internality, of which no item was amongst the top 27.

**Table 3:** Item ranking in terms of correlation with total score.

Rank	1	2	3	4	5	6	7	8	9	10	11	12	13	14
Scenario	7	7	5	2	4	3	7	6	2	3	6	7	6	7
Item	7	4	4	2	7	4	5	5	9	5	3	2	2	9
Rank	15	16	17	18	19	20	21	22	23	24	25	26	27	
Scenario	4	6	5	6	3	2	4	5	5	5	4	3	2	
Item	2	9	8	8	3	3	9	7	9	5	8	9	8	

The 27 items could also be used to estimate the separate subscales (other than internality). The correlation between the globality score measured on the full dataset and measured with the reduced set of 27 items was 0.68 (*p* = 10^−29^). Similarly, the correlations for stability and self-worth were 0.71 and 0.72 (both *p* < 10^−30^) and for negative consequences 0.49 (*p* = 4×10^−6^).

A PCA still identified a single underlying factor (Fig 1D) explaining 32% of the variance with positive loadings for all 27 items. The scores on the respective main PCA factors were highly correlated in the full and reduced item sets (correlation of 0.98, *p* < 10^−130^).

The 27 items were used to define a new, very short form (VSF) of the CSQ. This CSQ-VSF-D is included in S3 File.

### 3.2 Subscales

Subscales and total variability were also reliably related (*α* = 0.78), which is very similar to the results of the English version [9]. When considering how individual items related to the subscales, Cronbach’s *α* ranged from good (*α* = 0.82 for self-worth) to poor (*α* = 0.48 for internality). A PCA across components also indicated the existence of a single underlying factor explaining 65% of the variance (Fig 1B). The subscales all loaded positively on the main component (loadings between 0.13 and 0.69). A factor analysis across the subscales also indicated the existence of a single underlying factor (BIC = 53.4 for one vs BIC = 74.6 for two factors).

We repeated the analysis from Fig 1D using subscales instead of individual items. Fig 1E shows that the most informative subscale was ‘negative consequences’, followed by ‘globality’ and ‘stability’. These three together resulted in a summed score that had a high correlation (0.92) with the total score.

### 3.3 Scenarios

The reliability across scenarios was good (*α* = 0.86). Principal component analysis again indicated the presence of one single underlying factor (Fig 1C) explaining 53% of the variance. All scenarios loaded positively (range 0.18–0.44) onto this main eigenvector.

Repeating the analysis of Fig 1D, a correlation of 0.95 with the total score could be achieved by including scenarios 3, 5, 8, 2 and 7.

### 3.4 External validity

As a measure of external validity, we collected additional measures in a convenience subset of 90 subjects (see methods). Depressive symptoms were assessed using the Inventory of Depressive Symptomatology (IDS-SR 30; [20]). Table 4 shows that there were significant correlations with all subscales of the CSQ-SF-D, with the highest correlation being observed for the total score. Again, the internality subscale differed from the other subscales and had a smaller correlation with depressive symptomatology. A similar pattern is observed for correlation with state and trait anxiety (measured with the Spielberger State and Trait Anxiety (STAI) questionnaire [17]). Furthermore, CSQ-SF-D subscales and total scores correlated positively with punishment sensitivity, but not with reward sensitivity measured via the Sensitivity to Punishment and Sensitivity to Reward Questionnaire [16]). Interestingly, though, there was no correlation between punishment sensitivity on the SPSRQ and the CSF-SF-D subscale ‘negative consequences’.

**Table 4:** Correlation between total score and subscales with external measures of depression (IDS-SR 30), state and trait anxiety (STAI-S and STAI-T) and reward and punishment sensitivity. Total CSQ-SF-D scores are highly significantly correlated with measures of anxiety, depression and punishment sensitivity, but not reward sensitivity. Boldface indicates significant Pearson’s linear correlations.

		Internality	Globality	Stability	SelfWorth	NegCons	Total
IDS-SR 30	correlation	**0.21**	**0.41**	**0.37**	**0.4**	**0.32**	**0.46**
	p-value	0.04	5×10^−5^	0.0003	0.0001	0.002	6×10^−6^
STAI-S	correlation	0.17	**0.54**	**0.37**	**0.38**	**0.44**	**0.49**
	p-value	0.11	3×10^−8^	0.0004	0.0002	1×10^−5^	9×10^−7^
STAI-T	Correlation	0.24	**0.47**	**0.37**	**0.42**	**0.28**	**0.47**
	p-value	0.02	3×10^−6^	0.0004	3×10^−5^	0.007	2×10^−6^
Punishment Sens.	Correlation	0.23	**0.49**	**0.41**	**0.35**	0.16	**0.44**
	p-value	0.03	1×10^−6^	5×10^−5^	0.0007	0.13	1×10^−5^
Reward Sens.	Correlation	0.14	0.01	-0.01	0.05	0.12	0.07
	p-value	0.18	0.95	0.96	0.63	0.25	0.52

## 4 Discussion

We here provide a German translation of the short version of the Cognitive Style Questionnaire (CSQ-SF) by [9] with a validation. The translation included back-translation and was performed by multiple qualified clinicians fluent in both English and German. In order to render the questionnaire outside the university setting, two items were slightly modified to a continuing education rather than a university/school setting. The resulting German version has similar overall measures of reliability and also measures one single underlying factor.

The resulting scale has an internal reliability of *α* = 0.91 comparable to that of the English version with an *α* = 0.85. The German and English versions also have a similar factor structure, with one dominant dimension loading positively on most items; on all subscales; and on all scenarios. The first factor explained numerically the same amount (65%) of the variance in the five subscales of the English and the translated German version.

When examining the various components of the questionnaire, the measure of internality appears less robust than the others. This is again akin to the results in the 11 and 13-item versions of [9], though in the 8-item version that forms the basis of the current translation this appeared to be less the case.

The CSF-SF-D has reasonable external validity in terms of correlations with depressive and anxiety symptoms. It also correlated with punishment sensitivity on the SPSRQ, but not with reward sensitivity. Again, the internality subscale appeared to also have less external validity in being less correlated with any of the external measures. The measures of external validity are comparable between the German and the original English versions. The correlation between the English CSQ-SF and the Hospital Anxiety and Depression Scale (HADS) Anxiety and Depression scales were 0.38 and 0.28, respectively. Here, we find correlations with STAI state and trait anxiety measures of 0.49 and 0.47, and with total scores of the IDS-SR-30, a measure of depression [20] of 0.46. Note that while these values are not directly comparable as we did not acquire the HADS, they do nevertheless speak to the question of external validity in a comparable manner.

Given that the scores were clearly well captured by a single dimension, we again raised the question whether it could be further simplified. Our item-based analysis suggested that a very short form (henceforth CSQ-VSF-D) with only 27 items should yield very similar results to the short form both in terms of total score, factor structure and subscales. The CSQ-VSF-D removes two scenarios (1—‘Imagine you are getting along badly with your parents’ and 8—’Imagine you are unhappy’) entirely and no longer measures the internality subscale. It is also provided in the appendix and remains to be validated separately.

Limitations of this study include, most prominently, that no patients with depression were studied, and that hence its validity in clinical populations remains to be tested. Second, the external validity measures were performed in a convenience sample rather than a separate unbiased sample. Finally, we were not able to examine test-retest validity. These issues should be addressed in future work.

## Supporting Information

S1 File German translation of the Cognitive Style Questionnaire short from (CSQ_SF) with 72 items. (PDF)

S2 File The raw data and a matlab analysis script for all reported analyses and that generates Fig 1 are contained in the supporting information file. (GZ)

S3 File German very short from of the Cognitive Style Questionnaire with 27 items. (PDF)
