# Supplementary material for: German Translation and Validation of the Cognitive Style Questionnaire Short Form (CSQ-SF-D)
Source: PLoS One. 2016 Mar 2;11(3):e0149530. doi: 10.1371/journal.pone.0149530 (PMC4774974; doi:10.1371/journal.pone.0149530)
Supplement: S1 File — (PDF) [file pone.0149530.s001.pdf]

Cognitive Style Questionnaire – Short Form German Version (CSQ-SF-DE)

Quentin J.M. Huys[1,2], Frederike Petzschner[1], Daniel Renz[1], Isabel Berwian[1,2] and Helene Haker[1]

1 Translational Neuromodeling Unit, Institute for Biomedical Engineering, University of Zürich and ETH Zürich; **2** Department of Psychiatry, Psychotherapy und Psychosomatics, Hospital of Psychiatry, University of Zürich

German translation of: Meins, E., McCarthy-Jones, S., Fernyhough, C., Lewis, G., Bentall, R. P., and Alloy, L. B. (2012). Assessing negative cognitive style: Development and validation of a short-form version of the Cognitive Style Questionnaire. *Personality and Individual Differences, 52,* 581-585.

Scoring Instructions

Higher scores on the CSQ-SF indicate more negative cognitive style. For all standard-scored items, scores are as follows:

Strongly agree = 5

Agree = 4

Undecided = 3

Disagree = 2

Strongly disagree = 1

Reverse-scored items are indicated on the CSQ-SF with an asterisk (*), and receive a score of 5 for 'strongly disagree', 4 for 'disagree', and so on. Possible CSQ-SF scores thus range between 72 and 360.

Internality sub-scale: Scores for items 1 and 6 can be totalled to give the overall score for internality. Possible scores thus range between 16 and 80.

Globality sub-scale: Scores for items 2 and 7 can be totalled to give the overall score for globality. Possible scores thus range between 16 and 80.

Stability sub-scale: Scores for items 3 and 8 can be totalled to give the overall score for stability. Possible scores thus range between 16 and 80.

Negative consequences sub-scale: Scores for item 4 can be totalled to give the overall score for negative consequences. Possible scores thus range between 8 and 40.

Self-worth implications sub-scale: Scores for items 5 and 9 can be totalled to give the overall score for self-worth implications. Possible scores thus range between 16 and 80.

Bitte versuchen Sie, sich lebhaft jede der nachfolgenden Situationen vorzustellen. Malen Sie sich jede Situation so deutlich wie möglich aus, als ob sie Ihnen gerade in diesem Moment passieren würde. Versetzen Sie sich in die Situation und entscheiden Sie, was die Situation Ihrem Empfinden nach **verursacht** hätte, wenn sie **Ihnen** tatsächlich passiert wäre. Die Vorfälle können vielerlei Ursachen haben. Wählen Sie nur diejenige, die nach Ihrem Gefühl die Hauptursache für die Situation wäre, wenn sich die Situation in Ihrem Leben ereignet hätte.

Es gibt **keine richtigen oder falschen Antworten**. Beantworten Sie die Fragen so, dass Ihre Antworten dem entsprechen, was Sie selbst in der Situation denken und fühlen würden.

1. Stellen Sie sich vor, dass Sie sich schlecht mit Ihren Eltern verstehen.

Denken Sie sorgfältig über den Grund dafür nach, weshalb Sie sich schlecht mit Ihren Eltern verstehen. Beantworten Sie erst danach die folgenden Fragen.

	Trifft völlig zu	Trifft zu	Unklar	Trifft nicht zu	Trifft gar nicht zu
1. Dass ich mich schlecht mit meinen Eltern verstehe liegt an den Umständen oder an anderen Menschen. *	☐	☐	☐	☐	☐
2. Der Grund dafür, dass ich mich schlecht mit meinen Eltern verstehe, führt zu Problemen in allen Bereichen meines Lebens.	☐	☐	☐	☐	☐
3. Meine Eltern und ich werden einen Neuanfang setzen und den Grund vergessen, warum wir uns schlecht verstehen. *	☐	☐	☐	☐	☐
4. Dass ich mich schlecht mit meinen Eltern verstehe wird dazu führen, dass mir andere negative Dinge zustoßen werden.	☐	☐	☐	☐	☐
5. Dass ich mich schlecht mit meinen Eltern verstehe bedeutet, dass etwas mit mir als Person nicht stimmt.	☐	☐	☐	☐	☐
6. Es ist meine Schuld, dass ich mich schlecht mit meinen Eltern verstehe.	☐	☐	☐	☐	☐
7. Der Grund dafür, weshalb ich mich schlecht mit meinen Eltern verstehe, hindert mich nicht daran, andere Dinge zu genießen. *	☐	☐	☐	☐	☐
8. Der Grund, weshalb ich mich schlecht mit meinen Eltern verstehe wird mich auch in Zukunft daran hindern, mich mit meinen Eltern zu verstehen.	☐	☐	☐	☐	☐
9. Dass ich mich schlecht mit meinen Eltern verstehe sagt nichts über mich als Person aus. *	☐	☐	☐	☐	☐

2. Stellen Sie sich vor, dass die Zuhörer negativ auf einen wichtigen Vortrag reagieren, den Sie als Teil einer Fortbildung halten müssen.

Denken Sie sorgfältig über den Grund dafür nach, weshalb die Zuhörer schlecht auf Ihren Vortrag reagiert haben. Beantworten Sie erst danach die folgenden Fragen.

	Trifft völlig zu	Trifft zu	Unklar	Trifft nicht zu	Trifft gar nicht zu
1. Es ist nicht meine Schuld, dass die Zuhörer negativ reagiert haben. *	☐	☐	☐	☐	☐
2. Der Grund für die negative Reaktion der Zuhörer auf meinen Vortrag wird zu Versagen in allen Bereichen meines Lebens führen.	☐	☐	☐	☐	☐
3. Der Grund für die negative Reaktion der Zuhörer auf diesen Vortrag bedeutet, dass andere auch schlecht auf meine zukünftigen Vorträge reagieren werden.	☐	☐	☐	☐	☐
4. Die negativen Reaktionen auf meinen Vortrag werden nicht dazu führen, dass mir andere negative Dinge zustoßen werden. *	☐	☐	☐	☐	☐
5. Dass die Zuhörer negativ auf meinen Vortrag reagiert haben sagt nichts über mich als Person aus. *	☐	☐	☐	☐	☐
6. Dass die Zuhörer negativ auf meinen Vortrag reagiert haben liegt nicht an den Umständen oder anderen Menschen.	☐	☐	☐	☐	☐
7. Der Grund für die negativen Reaktionen trifft nur auf diesen Vortrag zu.*	☐	☐	☐	☐	☐
8. Der Grund für die negativen Reaktionen wird zukünftige Vorträge nicht beeinflussen.*	☐	☐	☐	☐	☐
9. Dass Leute negativ auf diesen Vortrag reagiert haben besagt eine Menge über mich als Person.	☐	☐	☐	☐	☐

3. Stellen Sie sich vor, dass Sie seit einem Jahr in dem von Ihnen erwünschen Job arbeiten. Sie erhalten jetzt eine schlechte Bewertung Ihrer Arbeitsleistung.

Denken Sie sorgfältig über den Grund dafür nach, weshalb Sie eine negative Bewertung erhalten haben. Beantworten Sie erst danach die folgenden Fragen.

	Trifft völlig zu	Trifft zu	Unklar	Trifft nicht zu	Trifft gar nicht zu
1. Dass ich eine schlechte Bewertung erhalten habe, liegt nicht an den Umständen oder anderen Menschen.	☐	☐	☐	☐	☐
2. Der Grund für die schlechte Bewertung trifft nur auf diese Bewertung meiner Arbeitsleistung zu. *	☐	☐	☐	☐	☐
3. Der Grund für meine schlechte Bewertung wird auch zukünftige Bewertungen meiner Arbeitsleistung beeinflussen.	☐	☐	☐	☐	☐
4. Diese schlechte Bewertung wird dazu führen, dass mir andere negative Dinge zustoßen werden.	☐	☐	☐	☐	☐
5. Diese schlechte Bewertung bedeutet, dass etwas mit mir als Person nicht stimmt.	☐	☐	☐	☐	☐
6. Es ist meine Schuld, dass ich diese schlechte Bewertung bekommen habe.	☐	☐	☐	☐	☐
7. Der Grund für diese schlechte Bewertung wird nicht zu Versagen in allen Bereichen meines Lebens führen. *	☐	☐	☐	☐	☐
8. Der Grund für diese schlechte Bewertung wird zukünftige Arbeitsbewertungen nicht beeinflussen. *	☐	☐	☐	☐	☐
9. Dass ich eine schlechte Bewertung bekommen habe besagt nichts über mich als Person. *	☐	☐	☐	☐	☐

4. Stellen Sie sich vor, Sie gehen auf eine Party und niemand interessiert sich für Sie.

Denken Sie sorgfältig über den Grund dafür nach, dass sich niemand für Sie interessiert. Beantworten Sie erst danach die folgenden Fragen.

	Trifft völlig zu	Trifft zu	Unklar	Trifft nicht zu	Trifft gar nicht zu
1. Es ist nicht meine Schuld, dass sich niemand für mich interessiert. *	☐	☐	☐	☐	☐
2. Der Grund dafür, dass sich niemand für mich interessiert, trifft nur auf diese Party zu.*	☐	☐	☐	☐	☐
3. Wenn ich in Zukunft auf eine ähnliche Party gehe, wird es anders kommen, und Leute werden sich für mich interessieren. *	☐	☐	☐	☐	☐
4. Dass sich auf dieser Party niemand für mich interessiert, wird nicht dazu führen, dass mir andere negative Dinge zustoßen werden. *	☐	☐	☐	☐	☐
5. Dass sich auf dieser Party niemand für mich interessiert, bedeutet nicht, dass etwas mit mir als Person nicht stimmt. *	☐	☐	☐	☐	☐
6. Der Grund, weshalb sich niemand für mich interessiert liegt nicht an anderen Menschen oder Ereignissen auf der Party.	☐	☐	☐	☐	☐
7. Der Grund dafür, dass sich auf dieser Party niemand für mich interessiert, wird zu Probleme in allen meinen Lebensbereiche führen.	☐	☐	☐	☐	☐
8. Der Grund, weshalb sich auf dieser Party niemand für mich interessierte, wird dazu führen, dass sich Leute auf zukünftigen Partys auch nicht für mich interessieren werden.	☐	☐	☐	☐	☐
9. Dass sich auf dieser Party niemand für mich interessierte besagt eine Menge über mich als Person.	☐	☐	☐	☐	☐

5. Stellen Sie sich vor, dass sie sich zutiefst wünschen, in einer intimen, romantischen Beziehung zu sein, es aber nicht sind.

Denken Sie sorgfältig über den Grund dafür nach, dass Sie nicht in einer Beziehung sind. Beantworten Sie erst danach die folgenden Fragen.

	Trifft völlig zu	Trifft zu	Unklar	Trifft nicht zu	Trifft gar nicht zu
1. Dass ich nicht in einer intimen, romantischen Beziehung bin liegt an Umständen oder anderen Menschen. *	☐	☐	☐	☐	☐
2. Der Grund dafür, dass ich nicht in einer Beziehung bin, betrifft nur intime romantische Beziehungen. *	☐	☐	☐	☐	☐
3. Der Grund dafür, weshalb ich nicht in einer Beziehung bin wird keinen Einfluss auf meine zukünftigen Beziehungen haben. *	☐	☐	☐	☐	☐
4. Weil ich nicht in einer intimen, romantischen Beziehung bin, werden mir andere negative Dinge widerfahren.	☐	☐	☐	☐	☐
5. Nicht in einer intimen, romantischen Beziehung zu sein bedeutet, dass mit mir als Person etwas nicht stimmt.	☐	☐	☐	☐	☐
6. Es ist meine Schuld, dass ich nicht in einer intimen, romantischen Beziehung bin.	☐	☐	☐	☐	☐
7. Der Grund, weshalb ich nicht in einer Beziehung bin führt zu Problemen in allen Bereichen meines Lebens.	☐	☐	☐	☐	☐
8. Der Grund, weshalb ich nicht in einer Beziehung bin bedeutet, dass ich auch in Zukunft keine intime, romantische Beziehung haben werde.	☐	☐	☐	☐	☐
9. Dass ich nicht in einer intimen, romantischen Beziehung bin besagt nichts über mich als Person. *	☐	☐	☐	☐	☐

6. Stellen Sie sich vor, dass Sie in einer wichtigen Fortbildung nicht alle Aufgaben erledigen können, die von Ihnen erwartet werden.

Denken Sie sorgfältig über den Grund dafür nach, weshalb Sie es nicht schaffen, die Aufgaben zu erledigen. Beantworten Sie erst danach die folgenden Fragen.

	Trifft völlig zu	Trifft zu	Unklar	Trifft nicht zu	Trifft gar nicht zu
1. Es ist nicht meine Schuld, dass ich es nicht geschafft habe, alle Aufgaben zu erledigen. *	☐	☐	☐	☐	☐
2. Der Grund, weshalb ich es nicht geschafft habe, alle Aufgaben zu erledigen, wird zu Problemen in allen Bereichen meines Lebens führen.	☐	☐	☐	☐	☐
3. Der Grund, weshalb ich es nicht geschafft habe, die Aufgaben zu erledigen, wird bei zukünftigen Fortbildungen zu ähnlichem Versagen beim Erledigen von Aufgaben führen.	☐	☐	☐	☐	☐
4. Nicht alle Aufgaben erledigt zu haben wird nicht dazu führen, dass mir andere negative Dinge zustoßen werden. *	☐	☐	☐	☐	☐
5. Dass ich diese Aufgaben nicht erledigen konnte besagt eine Menge über mich als Person.	☐	☐	☐	☐	☐
6. Dass ich es nicht geschafft habe, die Aufgaben zu erledigen, liegt an den Umständen oder an anderen Menschen.*	☐	☐	☐	☐	☐
7. Der Grund, weshalb ich die Aufgaben nicht erledigen konnte, trifft nur auf diese Fortbildung zu. *	☐	☐	☐	☐	☐
8. Der Grund weshalb ich es nicht geschafft habe, alle Aufgaben zu erledigen wird nicht beeinflussen, ob ich in zukünftigen Fortbildungen die Aufgaben erledigen werde.*	☐	☐	☐	☐	☐
9. Dass ich die Aufgaben nicht erledigt habe besagt nichts über mich als Person. *	☐	☐	☐	☐	☐

7. Stellen Sie sich vor, dass eine Person, mit der Sie gerne eng befreundet wären, nicht mit Ihnen befreundet sein möchte.

Denken Sie sorgfältig über den Grund dafür nach, weshalb die Person nicht mit Ihnen befreundet sein möchte. Beantworten Sie erst danach die folgenden Fragen.

	Trifft völlig zu	Trifft zu	Unklar	Trifft nicht zu	Trifft gar nicht zu
1. Dass diese Person nicht mit mir befreundet sein möchte liegt an den Umständen oder an anderen Menschen. *	☐	☐	☐	☐	☐
2. Der Grund, weshalb diese Person nicht mit mir befreundet sein möchte, betrifft nur diese eine Freundschaft. *	☐	☐	☐	☐	☐
3. Der Grund, weshalb diese Person nicht mit mir befreundet sein möchte, wird diese Person immer davon abhalten, sich mit mir anzufreunden.	☐	☐	☐	☐	☐
4. Dass diese Person nicht mit mir befreundet sein möchte wird dazu führen, dass mir andere negative Dinge widerfahren.	☐	☐	☐	☐	☐
5. Dass diese Person nicht mit mir befreundet sein möchte bedeutet, dass etwas mit mir als Person nicht stimmt.	☐	☐	☐	☐	☐
6. Es ist meine Schuld, dass diese Person nicht mit mir befreundet sein möchte.	☐	☐	☐	☐	☐
7. Der Grund dafür, dass diese Person nicht mit mir befreundet sein möchte, führt zu Problemen in allen Bereichen meines Lebens.	☐	☐	☐	☐	☐
8. Diese Person könnte anders über den Grund urteilen, weswegen sie nicht mit mir befreundet ist, und in Zukunft mein Freund werden. *	☐	☐	☐	☐	☐
9. Dass diese Person nicht mit mir befreundet sein möchte, besagt nichts über mich als Person. *	☐	☐	☐	☐	☐

8. Stellen Sie sich vor, dass Sie unglücklich sind.

Denken Sie sorgfältig über den Grund dafür nach, dass Sie unglücklich sind. Beantworten Sie erst danach die folgenden Fragen.

	Trifft völlig zu	Trifft zu	Unklar	Trifft nicht zu	Trifft gar nicht zu
1. Dass ich unglücklich bin liegt an den Umständen oder an anderen Menschen. *	☐	☐	☐	☐	☐
2. Der Grund, weshalb ich unglücklich bin, beeinträchtigt nur meine Stimmung. *	☐	☐	☐	☐	☐
3. Der Grund, weshalb ich unglücklich bin, wird immer dazu führen, dass ich unglücklich sein werde.	☐	☐	☐	☐	☐
4. Unglücklich zu sein wird nicht dazu führen, dass mir andere negative Dinge zustoßen. *	☐	☐	☐	☐	☐
5. Unglücklich zu sein bedeutet nicht, dass mit mir als Person etwas nicht stimmt. *	☐	☐	☐	☐	☐
6. Es ist meine Schuld, dass ich unglücklich bin.	☐	☐	☐	☐	☐
7. Der Grund, weshalb ich unglücklich bin, führt zu Problemen in allen meinen Bereichen meines Lebens.	☐	☐	☐	☐	☐
8. Der Grund, weshalb ich unglücklich bin, wird vorübergehen und künftig nie mehr dazu führen, dass ich unglücklich sein werde. *	☐	☐	☐	☐	☐
9. Unglücklich zu sein besagt eine Menge über mich als Person.	☐	☐	☐	☐	☐
