# Supplementary material for: German Translation and Validation of the Cognitive Style Questionnaire Short Form (CSQ-SF-D)
Source: PLoS One. 2016 Mar 2;11(3):e0149530. doi: 10.1371/journal.pone.0149530 (PMC4774974; doi:10.1371/journal.pone.0149530)
Supplement: S3 File — (PDF) [file pone.0149530.s003.pdf]

Cognitive Style Questionnaire – Very Short Form German Version (CSQ-VSF-DE)

Quentin J.M. Huys[1,2], Frederike Petzschner[1], Daniel Renz[1], Isabel Berwian[1,2] and Helene Haker[1]

1 Translational Neuromodeling Unit, Institute for Biomedical Engineering, University of Zürich and ETH Zürich; **2** Department of Psychiatry, Psychotherapy und Psychosomatics, Hospital of Psychiatry, University of Zürich

German translation and shortened version of: Meins, E., McCarthy-Jones, S., Fernyhough, C., Lewis, G., Bentall, R. P., and Alloy, L. B. (2012). Assessing negative cognitive style: Development and validation of a short-form version of the Cognitive Style Questionnaire. *Personality and Individual Differences, 52,* 581-585*.*

Scoring Instructions

Higher scores on the CSQ-VSF indicate more negative cognitive style. For all standard-scored items, scores are as follows:

Strongly agree = 5

Agree = 4

Undecided = 3

Disagree = 2

Strongly disagree = 1

Reverse-scored items are indicated on the CSQ-SF with an asterisk (*), and receive a score of 5 for 'strongly disagree', 4 for 'disagree', and so on. Possible CSQ-SF scores thus range between 27 and 135.

Globality sub-scale: Sum Scenario 1 item 1, Scenario 3 items 1 and 2, Scenario 4 item 3, Scenario 5 item 1, Scenario 6 items 1 and 4.

Stability sub-scale: Sum Scenario 1 items 2 & 3, Scenario 2 item 1, Scenario 3 item 3, Scenario 4 item 4, Scenario 5 items 2 & 4.

Negative consequences sub-scale: Sum Scenario 1 item 4, Scenario 2 items 3 & 4, Scenario 3 item 4, Scenario 4 items 2 and 5, Scenario 5 items 3 & 5, Scenario 6 items 3 & 5

Self-worth implications sub-scale: Sum Scenario 2 item 2, Scenario 4 item 1, Scenario 6 item 2

Bitte versuchen Sie, sich lebhaft jede der nachfolgenden Situationen vorzustellen. Malen Sie sich jede Situation so deutlich wie möglich aus, als ob sie Ihnen gerade in diesem Moment passieren würde. Versetzen Sie sich in die Situation und entscheiden Sie, was die Situation Ihrem Empfinden nach **verursacht** hätte, wenn sie **Ihnen** tatsächlich passiert wäre. Die Vorfälle können vielerlei Ursachen haben. Wählen Sie nur diejenige, die nach Ihrem Gefühl die Hauptursache für die Situation wäre, wenn sich die Situation in Ihrem Leben ereignet hätte.

Es gibt **keine richtigen oder falschen Antworten**. Beantworten Sie die Fragen so, dass Ihre Antworten dem entsprechen, was Sie selbst in der Situation denken und fühlen würden.

1. **Stellen Sie sich vor, dass die Zuhörer negativ auf einen wichtigen Vortrag reagieren, den Sie als Teil einer Fortbildung halten müssen.**

Denken Sie sorgfältig über den Grund dafür nach, weshalb die Zuhörer schlecht auf Ihren Vortrag reagiert haben. Beantworten Sie erst danach die folgenden Fragen.

	Trifft völlig zu	Trifft zu	Unklar	Trifft nicht zu	Trifft gar nicht zu
1. Der Grund für die negative Reaktion der Zuhörer auf meinen Vortrag wird zu Versagen in allen Bereichen meines Lebens führen.	☐	☐	☐	☐	☐
2. Der Grund für die negative Reaktion der Zuhörer auf diesen Vortrag bedeutet, dass andere auch schlecht auf meine zukünftigen Vorträge reagieren werden.	☐	☐	☐	☐	☐
3. Der Grund für die negativen Reaktionen wird zukünftige Vorträge nicht beeinflussen.*	☐	☐	☐	☐	☐
4. Dass Leute negativ auf diesen Vortrag reagiert haben besagt eine Menge über mich als Person.	☐	☐	☐	☐	☐

2. Stellen Sie sich vor, dass Sie seit einem Jahr in dem von Ihnen erwünschen Job arbeiten. Sie erhalten jetzt eine schlechte Bewertung Ihrer Arbeitsleistung.

Denken Sie sorgfältig über den Grund dafür nach, weshalb Sie eine negative Bewertung erhalten haben. Beantworten Sie erst danach die folgenden Fragen.

	Trifft völlig zu	Trifft zu	Unklar	Trifft nicht zu	Trifft gar nicht zu
1. Der Grund für meine schlechte Bewertung wird auch zukünftige Bewertungen meiner Arbeitsleistung beeinflussen.	☐	☐	☐	☐	☐
2. Diese schlechte Bewertung wird dazu führen, dass mir andere negative Dinge zustoßen werden.	☐	☐	☐	☐	☐
3. Diese schlechte Bewertung bedeutet, dass etwas mit mir als Person nicht stimmt.	☐	☐	☐	☐	☐
4. Dass ich eine schlechte Bewertung bekommen habe besagt nichts über mich als Person. *	☐	☐	☐	☐	☐

3. Stellen Sie sich vor, Sie gehen auf eine Party und niemand interessiert sich für Sie.

Denken Sie sorgfältig über den Grund dafür nach, dass sich niemand für Sie interessiert. Beantworten Sie erst danach die folgenden Fragen.

	Trifft völlig zu	Trifft zu	Unklar	Trifft nicht zu	Trifft gar nicht zu
1. Der Grund dafür, dass sich niemand für mich interessiert, trifft nur auf diese Party zu.*	☐	☐	☐	☐	☐
2. Der Grund dafür, dass sich auf dieser Party niemand für mich interessiert, wird zu Probleme in allen meinen Lebensbereiche führen.	☐	☐	☐	☐	☐
3. Der Grund, weshalb sich auf dieser Party niemand für mich interessierte, wird dazu führen, dass sich Leute auf zukünftigen Partys auch nicht für mich interessieren werden.	☐	☐	☐	☐	☐
4. Dass sich auf dieser Party niemand für mich interessierte besagt eine Menge über mich als Person.	☐	☐	☐	☐	☐

4. Stellen Sie sich vor, dass sie sich zutiefst wünschen, in einer intimen, romantischen Beziehung zu sein, es aber nicht sind.

Denken Sie sorgfältig über den Grund dafür nach, dass Sie nicht in einer Beziehung sind. Beantworten Sie erst danach die folgenden Fragen.

	Trifft völlig zu	Trifft zu	Unklar	Trifft nicht zu	Trifft gar nicht zu
1. Weil ich nicht in einer intimen, romantischen Beziehung bin, werden mir andere negative Dinge widerfahren.	☐	☐	☐	☐	☐
2. Nicht in einer intimen, romantischen Beziehung zu sein bedeutet, dass mit mir als Person etwas nicht stimmt.	☐	☐	☐	☐	☐
3. Der Grund, weshalb ich nicht in einer Beziehung bin führt zu Problemen in allen Bereichen meines Lebens.	☐	☐	☐	☐	☐
4. Der Grund, weshalb ich nicht in einer Beziehung bin bedeutet, dass ich auch in Zukunft keine intime, romantische Beziehung haben werde.	☐	☐	☐	☐	☐
5. Dass ich nicht in einer intimen, romantischen Beziehung bin besagt nichts über mich als Person. *	☐	☐	☐	☐	☐

5. Stellen Sie sich vor, dass Sie in einer wichtigen Fortbildung nicht alle Aufgaben erledigen können, die von Ihnen erwartet werden.

Denken Sie sorgfältig über den Grund dafür nach, weshalb Sie es nicht schaffen, die Aufgaben zu erledigen. Beantworten Sie erst danach die folgenden Fragen.

	Trifft völlig zu	Trifft zu	Unklar	Trifft nicht zu	Trifft gar nicht zu
1. Der Grund, weshalb ich es nicht geschafft habe, alle Aufgaben zu erledigen, wird zu Problemen in allen Bereichen meines Lebens führen.	☐	☐	☐	☐	☐
2. Der Grund, weshalb ich es nicht geschafft habe, die Aufgaben zu erledigen, wird bei zukünftigen Fortbildungen zu ähnlichem Versagen beim Erledigen von Aufgaben führen.	☐	☐	☐	☐	☐
3. Dass ich diese Aufgaben nicht erledigen konnte besagt eine Menge über mich als Person.	☐	☐	☐	☐	☐
4. Der Grund weshalb ich es nicht geschafft habe, alle Aufgaben zu erledigen wird nicht beeinflussen, ob ich in zukünftigen Fortbildungen die Aufgaben erledigen werde.*	☐	☐	☐	☐	☐
5. Dass ich die Aufgaben nicht erledigt habe besagt nichts über mich als Person. *	☐	☐	☐	☐	☐

6. Stellen Sie sich vor, dass eine Person, mit der Sie gerne eng befreundet wären, nicht mit Ihnen befreundet sein möchte.

Denken Sie sorgfältig über den Grund dafür nach, weshalb die Person nicht mit Ihnen befreundet sein möchte. Beantworten Sie erst danach die folgenden Fragen.

	Trifft völlig zu	Trifft zu	Unklar	Trifft nicht zu	Trifft gar nicht zu
1. Der Grund, weshalb diese Person nicht mit mir befreundet sein möchte, betrifft nur diese eine Freundschaft. *	☐	☐	☐	☐	☐
2. Dass diese Person nicht mit mir befreundet sein möchte wird dazu führen, dass mir andere negative Dinge widerfahren.	☐	☐	☐	☐	☐
3. Dass diese Person nicht mit mir befreundet sein möchte bedeutet, dass etwas mit mir als Person nicht stimmt.	☐	☐	☐	☐	☐
4. Der Grund dafür, dass diese Person nicht mit mir befreundet sein möchte, führt zu Problemen in allen Bereichen meines Lebens.	☐	☐	☐	☐	☐
5. Dass diese Person nicht mit mir befreundet sein möchte, besagt nichts über mich als Person. *	☐	☐	☐	☐	☐
